# Putative histidine kinase inhibitors with antibacterial effect against multi-drug resistant clinical isolates identified by *in vitro* and *in silico* screens

**DOI:** 10.1038/srep26085

**Published:** 2016-05-13

**Authors:** Nadya Velikova, Simone Fulle, Ana Sousa Manso, Milena Mechkarska, Paul Finn, J. Michael Conlon, Marco Rinaldo Oggioni, Jerry M. Wells, Alberto Marina

**Affiliations:** 1 Instituto de Biomedicina de Valencia, Consejo Superior de Investigaciones Científicas (CSIC), Jaume Roig 11, 46010 Valencia, Spain; 2 InhibOx Limited, Oxford, OX1 1BY, United Kingdom; 3 Dipartimento di Biotecnologie Mediche, Universita di Siena, 53100 Siena, Italy; 4 Department of Genetics, University of Leicester, Leicester, Le1 7RH, United Kingdom; 5 Department of Biochemistry, College of Medicine and Health Science, United Arab Emirates Univesity, P.O. Box 17666 Al Ain, United Arab Emirates; 6 Host-Microbe Interactomics Chair Group, Animal Sciences, University of Wageningen, P.O. Box 338, 6700 AH Wageningen, The Netherlands; 7 CIBER de Enfermedades Raras (CIBERER), ISCIII, Valencia, Spain

## Abstract

Novel antibacterials are urgently needed to address the growing problem of bacterial resistance to conventional antibiotics. Two-component systems (TCS) are widely used by bacteria to regulate gene expression in response to various environmental stimuli and physiological stress and have been previously proposed as promising antibacterial targets. TCS consist of a sensor histidine kinase (HK) and an effector response regulator. The HK component contains a highly conserved ATP-binding site that is considered to be a promising target for broad-spectrum antibacterial drugs. Here, we describe the identification of putative HK autophosphorylation inhibitors following two independent experimental approaches: *in vitro* fragment-based screen via differential scanning fluorimetry and *in silico* structure-based screening, each followed up by the exploration of analogue compounds as identified by ligand-based similarity searches. Nine of the tested compounds showed antibacterial effect against multi-drug resistant clinical isolates of bacterial pathogens and include three novel scaffolds, which have not been explored so far in other antibacterial compounds. Overall, putative HK autophosphorylation inhibitors were found that together provide a promising starting point for further optimization as antibacterials.

Bacterial multi-drug resistance (MDR) is defined as acquisition by pathogenic bacteria of non-susceptibility to at least one agent in three categories of antibacterials^1^. MDR is a growing problem worldwide^2^ and has led the World Health Organization (WHO) to classify antibacterial resistance and the antibiotics crisis to be a health problem “bigger than AIDS”. The so-called “ESKAPE” pathogens (*Enterococcus faecium, Staphylococcus aureus, Klebsiella pneumonia, Acinetobacter baumannii, Pseudomonas aeruginosa, Enterobacter spp*.) are the main cause of hospital infections and are resistant to virtually all currently marketed antibiotics^3^. The aging population and growing number of immunocompromised patients due to HIV, cancer therapy or transplantation have increased the population susceptibility to bacterial infections making the need for novel antibacterials even more acute. At the same time, MDR represent an economical problem since infections due to resistant bacteria have 1.3–2 fold higher associated healthcare costs than susceptible bacteria due to increased mortality, morbidity and treatment costs^4^.

Bacterial two-component systems (TCS) have been proposed as promising targets for the discovery of novel antibacterials with a new mechanism of action and with lower potential of resistance development in comparison with conventional antibiotics^5^,^6^. TCS are signal transduction devices used by nearly all bacteria that regulate a variety of processes including bacterial growth, cell-wall metabolism, virulence, biofilm formation and resistance to antibiotics^7^,^8^. A prototypical TCS consist of a membrane bound histidine kinase (HK) and its cognate response regulator (RR). Upon sensing an environmental stimulus the HK is autophosphorylated on conserved histidine residues in the dimerization and histidine phosphotransfer (DHp) domain by an ATP molecule binding to the catalytic and ATP-binding (CA) domain. Subsequently, the phosphoryl group from the His is transferred to a conserved aspartic acid residue in the receiver (REC) domain of the RR. The phosphorylated state of the RR affects its binding affinity to a cognate DNA motif and/or other protein partners, thereby modulating transcription of target genes^8^. HK autophosphorylation inhibitors (HKAIs) targeted at the CA domains of HKs are expected and were recently shown to simultaneously inhibit multiple TCS due to the conservation between the HK CA domains^6^,^9^. Furthermore, the CA domain fold of the HKs is completely different to the fold found in mammalian Ser/Thr/Tyr kinases^10^,^8^, providing the inhibitors targeting CA domain selectivity for HKs and reducing the probability of potential side effects.

So far, TCS inhibitors have been discovered mainly by *in vitro* high-throughput screening (HTS)^11^,^12^,^13^ or by structure-based virtual screening (SBVS) experiments^14^,^15^,^16^,^17^,^18^. SBVS is nowadays an indispensable component within drug discovery efforts, including hit identification and optimization^14^,^15^,^16^,^17^,^18^,^19^,^20^,^21^,^22^. Alternatively, fragment-based screening (FBS) has become increasingly popular over the last 10 years because it allows an efficient exploration of chemical space and results into smaller hit compounds, which can be later optimized (e.g. regarding affinity or physicochemical properties)^23^,^24^,^25^. FBS can be done, for example, by soaking experiments via X-ray crystallography or by differential scanning fluorimetry (DSF) where the change of denaturation temperature of a protein is monitored in different conditions, including the presence of low-molecular weight ligands^26^,^27^.

Here, we report a step-wise application of the two complementary screening approaches mentioned above, i.e. *in silico* screening of small molecules and *in vitro* FBS by DSF, to identify putative HKAIs. The resulting hits are further explored by analogue compounds, as identified by ligand-based similarity searches (LBSS) of a public repository database. Both approaches yielded molecules that were capable to inhibit different HKs *in vitro*, and which showed antibacterial activity against laboratory strains and, even more valuable, against MDR clinical isolates, including methicillin-resistant *Staphylococcus aureus* (MRSA).

## Results and Discussion

### Two putative fragment-like HKAIs identified by *in vitro* screening

**Figure 1:**
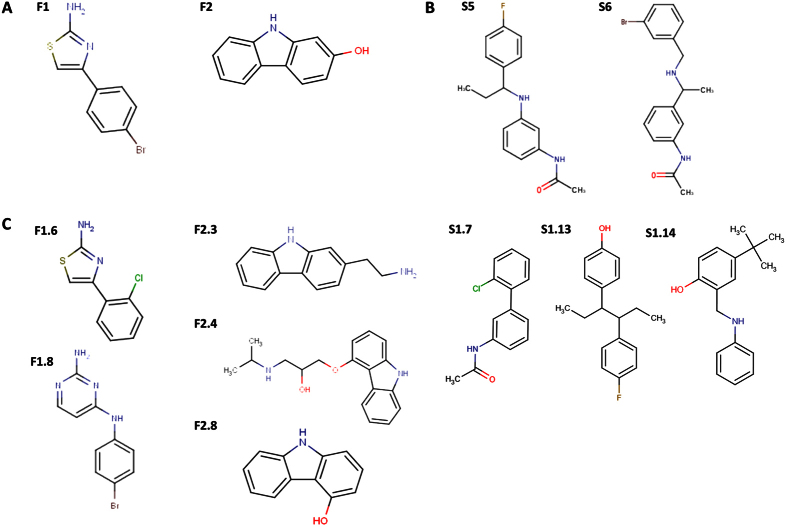
Chemical structures of selected HKAIs. (**A**) F1 and F2 were identified in an *in vitro* screening of a fragment library by differential scanning fluorimetry as putative ligands of HK CA domain (Fig. S1A). (**B**) S5 and S6 were identified in a SBVS for putative ligands of the CA domain of multiple HKs (Fig. S1B). (**C**) F1, F2, S5, and S6 were used as query molecules in ligand-based similarity searches (Fig. S1C) and F1.6, F1.8, F2.3, F2.4, F2.8, S1.7, S1.13, and S1.14 were among the hits showing promising *in vitro* activities (Tables 1 and 2).

**Figure 2:**
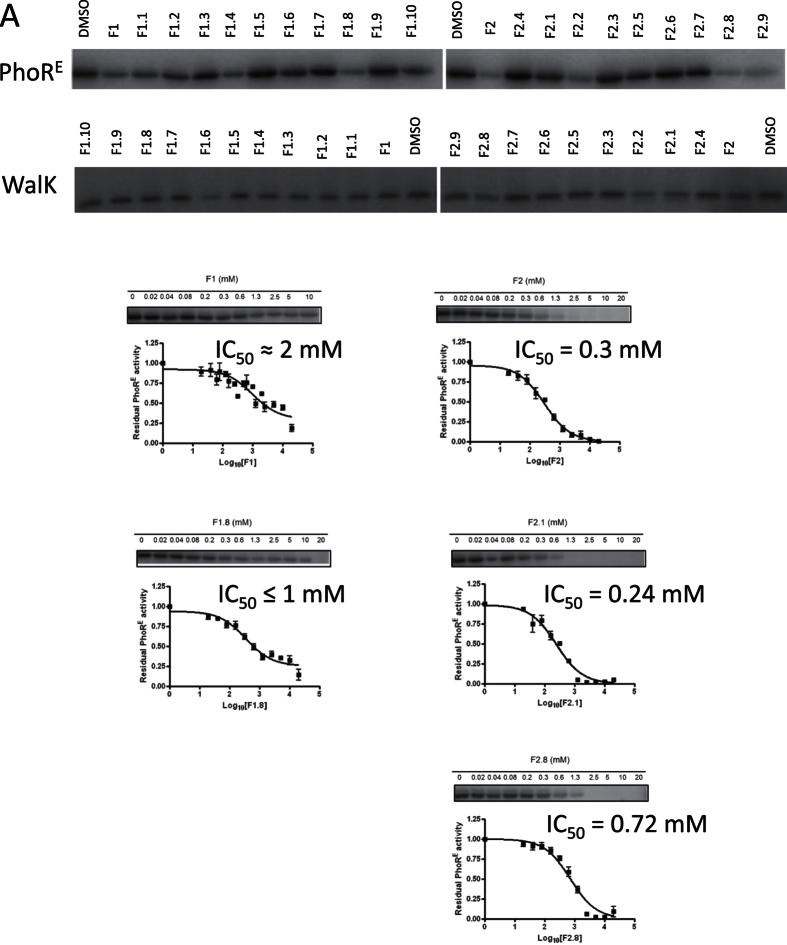
Biochemical evaluation by *in vitro* kinase assay of the FBS hits (F1 and F2) and their analogues (F1.1 to F1.9, and F2.1 to F2.10). (**A**) First, the autophosphorylation inhibitory activity was evaluated in a one concentration (2mM) one time-point (30 sec) *in vitro* kinase assays with WalK and PhoR^E^. The fragments inhibited WalK and PhoR^E^ autophosphorylation with 10 to 62% and 17 to 80%, respectively. (**B**) IC_50_ of the more potent inhibitors (% inhibition at 2 mM > 50%) with antibacterial effect were measured in a multiple-concentrations one time-point (30 sec) experiments.

To identify compounds with broad capacity to inhibit HK autophosphorylation we targeted the catalytic domain of HKs following two approaches. First, 898 fragment-like ligands (MW < 300, ClogP <3, number of hydrogen bond donors and hydrogen bond acceptors < 3, number of rotatable bonds <3^28^) of the Fragment Library 1 from Chem-X-Infinity (Romanville, France) were screened for binding to the CA domains of HKs via differential scanning fluorimetry (DSF)^27^ (Figs S1 and S2). As targets, we selected the HKs of two essential TCS, WalK-WalR of *Streptococcus pneumonia*^29^ and NblS-RapB of *Synechococcus sp.* PCC 7942^30^ (Fig. S1A). The presence of 4-(4-bromophenyl)-1,3-thiazol-2-amine (F1, Fig. 1) and 2-hydroxy-carbazole (F2) increased the temperature at which HK NblS (CA domain) unfolds (Tm) by 2.1 and 2.2 °C, respectively, suggesting that F1 and F2 are ligands for the CA domain of NblS (Fig. S2). Encouragingly, the screening for ligands of HK WalK (DHp and CA domain) showed that F1 and F2 were also among the hits increasing WalK Tm. F1 and F2 increased WalK Tm by 4.5 and 3.9 °C, respectively (Fig. S2). To test the HK inhibitory capacity of these compounds we carried out autophosphorylation assays with the radiolabeled γ-32P-ATP substrate. Since fragments usually show low affinity for their targets^31^,^28^, the assays were performed at high compound concentration to minimize the probability of discarding potential inhibitors with weak binding capacity. In the autophosphorylation reaction the HK also works as substrate and it was observed for several HKs that the reaction reaches saturation in short time, even more due to the accumulation of the product ADP that has inhibitory activity^32^,^33^,^34^. Therefore, to assure the linearity of the autophosphorylation reaction in respect to time and to maximize the effect of the putative inhibitors we initially checked the inhibitory capacity of these fragments to a single and high concentration (5 mM) at one short time point (30 sec). The assays showed that F1 and F2 have a weak inhibitory capacity for the autophosphorylation activity of the screened catalytic portion of WalK. However, F1 and F2 inhibited the autophosphorylation of PhoR from the Gram-negative *Escherichia coli* (PhoR^E^), with IC_50_ ≈ 2 mM (the compound showed limited solubility in kinase buffer) and 0.3 mM, respectively (Table 1, Fig. 2) suggesting HK inhibitory activity. Furthermore, F1 and F2 showed antibacterial effect against the Gram-positive *S. aureus* DSM 20231 with minimal inhibitory concentrations (MIC) of 25 and 31 μg/ml, respectively (Tables 1 and 2). F1 showed also antibacterial effect against *S. epidermidis* DSM 20044 with a MIC of 4 μg/ml.

### Structure-based virtual screening reveals two drug-like putative HKAIs

The ATP-binding pocket of the CA domains of HKs has been previously used in SBVS^14^. In virtual screening, both ligand and receptor flexibility should be considered. While the consideration of the ligand flexibility is straightforward^35^,^36^, the consideration of the receptor flexibility remains a major challenge for docking calculations^37^,^38^. However, docking into an ensemble of receptor structures has been shown to be a valuable mean to cope implicit with the receptor flexibility^39^,^40^,^41^. With that in mind, to identify drug-like ligands (MW < 500, ClogP ≤ 5, number of hydrogen bond donors ≤ 5, number of hydrogen bond acceptors ≤ 10^42^) of the ATP-binding sites of multiple HK CA domains, a diverse set of 600 000 drug-like compounds was screened via *in silico* docking calculation using as target receptors the ATP-binding sites of the CA domains of three different HKs: *T. maritima* HK853 (PDB: 3DGE)^32^, *T. maritima* CheA (PDB: 1I58)^43^ and *G. stearothermophilus* KinB (PDB: 3D36)^44^ (Fig. S1B). These three CA domains were selected because the structures were solved in the presence of a nucleotide and re-docking of the cognate ligand was successful (RMSD docked *vs* experimental nucleotide of 1 Å for 3DGE and 3D36, and 3 Å for 1I58). Furthermore, virtual screening with the three HK structures, that have some sequence variability at the ATP-binding site, was expected to facilitate the identification of general HKAIs.

It has been shown to be valuable to rescore docking solutions by ligand efficiency (l.e.) metrics as well as more rigorous rescoring approaches such as free energy perturbation (FEP), molecular mechanics Poisson-Boltzmann surface area (MM-PBSA) or linear interaction energy (LIE)^45^,^46^,^47^. However, the application of default protocols for binding free energy calculations can also lead to only moderate ligand rankings because it is recommended to identify the best procedure in a specific case^48^. Such a validation step is clearly hindered for rather unexplored targets such as the HKs used in the *in silico* screening reported here. Therefore, the screened compounds were ranked based on the raw docking score, ChemPLP^49^, as well as ligand efficiency (l.e.)^50^. The top 100 docked compounds in common for the three HKs were then visually inspected and resulted in the selection of 10 compounds, S1–S10 (Table S1 and Fig. S3), for experimental testing.

**Figure 3:**
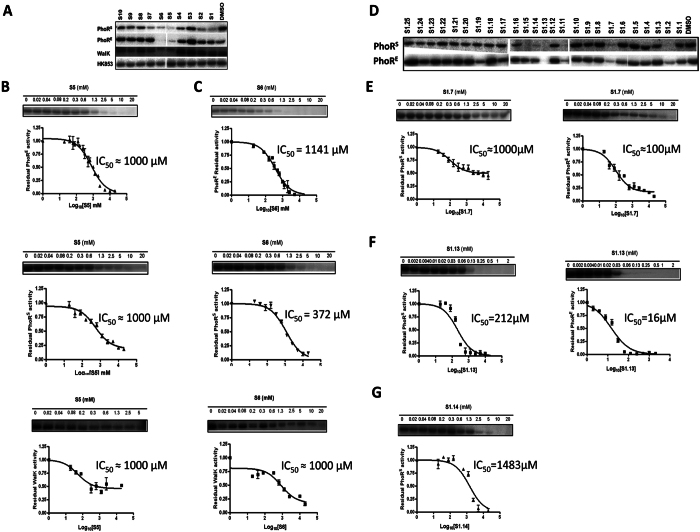
Autophosphorylation inhibitory activity of the initial hits from the SBVS (S1 to S10) and the analogues of S5 and S6, selected following LBSS. (**A**) One-time (30 sec) one-concentration (5 mM) kinase assay with the SBVS hits and PhoR^S^, PhoR^E^, WalK and HK853 distinguished S5 and S6 as relatively stronger multiple HKAIs. The IC_50_ for PhoR^S^, PhoR^E^ and WalK in presence of S5 (**B**) and S6 (**C**) were calculated from the autophosphorylation reaction assays at different concentrations of compounds. (**D**) One-time point (30 sec), one-concentration (2 mM) kinase assay with the LBSS hits and PhoR^S^ and PhoR^E^ HKs. The IC_50_ for PhoR^S^ and PhoR^E^ in presence of S1.7 (**E**), S1.13 (**F**) and S1.14 (**G**) were calculated from the autophosphorylation reaction assays at different concentrations of compounds. Error bars represent the standard errors of the mean (SEM) of two independent assays with two replicates.

The inhibitory activity of the 10 selected compounds from the SBVS on HK autophosphorylation was tested *in vitro* using four different HKs *T. maritima* HK853 (HK853), as a representative of the structures used in the docking assays, the highly extended HK PhoR from a Gram-negative (*E. coli*; PhoR^E^) and a Gram-positive (*S. aureus*; PhoR^S^) representative, and *S. pneumoniae* WalK (WalK) as a representative of the essential HKs^29^. As in the case of the fragments we performed initial *in vitro* kinase assays at a single and high compound concentration and at one time point (Fig. 3, Table 1). Autophosphorylation activity of HK853 was not or weakly (up to 30%) inhibited by the 10 compounds compared to the negative control (Fig. 3A) and it was not possible to identify more potent inhibitor(s) based on HK853 autophosphorylation inhibition. The kinase assays with WalK, PhoR^E^ and PhoR^S^ revealed that compounds S5 and S6 had a higher autophosphorylation inhibitory activity than the other selected compounds identified by SBVS and were general HKAIs (Fig. 3A, Table 1). S5 and S6 inhibit HK autophosphorylation activity in a dose-dependent manner, with IC_50_ in the high micromolar/ilimolar range (Fig. 3, Table 1). S5 inhibits PhoR^E^, PhoR^S^ and WalK with IC_50_ ≈ 1000 μM and it seems it is not soluble in kinase buffer in the presence of 10% DMSO at concentrations higher than 1.3 mM. S6 inhibits PhoR^E^, PhoR^S^ and WalK autophosphorylation with IC_50_ of 372 μM, 1141 μM and > 1000 μM, respectively (Fig. 3, Table 1).

Furthermore, evaluation of the antibacterial effect of the 10 selected compounds from SBVS for the Gram-positive (S*. aureus* DSM 20231 and *S. epidermidis* DSM 20044) and Gram-negative (*E. coli* UCF 073) bacteria, revealed that only S5 and S6 were able to inhibit bacterial growth at high compound concentrations, which is in agreement with their inhibitory activity on HKs (Table S2). *S. epidermidis* DSM 20044 growth was inhibited by S5 and S6 while *E. coli* UCF 073 growth was only inhibited by S6, in all the cases with modest MICs of 500 μg/ml that are in line with the low HK affinity suggested by the measured IC_50_ (Fig. 3, Table 1). Both compounds were bacteriostatic against the three bacterial strains in the tested concentration range (MBC > 500 μg/ml).

### Ligand-based similarity searches identified more potent HKAIs with stronger antibacterial activity

It is a well-accepted assumption that similar compounds have similar activity; however, small structural changes in a compound can result in significant difference in potency (so called ‘activity cliffs’)^51^. The latter is exemplified by S5 and S6 since the two compounds are structurally similar but they show different inhibitory capacity toward the HKs assayed (Figs S1 and 1). On the basis of the reported initial results (i.e. *in vitro* and *in silico* screenings, biochemical enzyme inhibition and antibacterial susceptibility testing) and attempting to identify more potent HKAIs with stronger antibacterial effect, analogue compounds of F1, F2, S5 and S6 (Fig. 1) in the database from the Developmental Therapeutics Program of the National Cancer Institute and the National Institute of Health (DTP) were identified using LBSS (i.e. by circular topological fingerprints as implemented in RDKit)^52^,^53^,^54^. The top 100 hits for the LBSS with the initial hits, F1, F2, S5 or S6 were visually investigated and a pool of 42 compounds (i.e. F1.1–F1.10, F2.1–F2.9, and S1.1–S1.25; Figs S1C, S4 and S5, Table S2) were experimentally evaluated for their HK autophosphorylation inhibitory capacity and antibacterial effect *in vitro.*

First, inhibition of autophosphorylation was measured at a single time point (30 sec) using one concentration (2 mM) of each putative inhibitor. PhoR^S^ and PhoR^E^ were used as targets for the analogues of S5 and S6 since these HKs were more strongly inhibited by S5 and S6 compared to WalK and HK853. Compounds S1.1 to S1.25 (2 mM) inhibited PhoR^E^ and PhoR^S^ autophosphorylation activity compared to the negative control from 6 to 85% and from 0 to 100%, respectively (Fig. 3 and Table S3). S1.2, S1.11, S1.13 inhibit autophosphorylation activity of both PhoR^S^ and PhoR^E^ with more than 75%. S1.7, S1.14 and S1.15 inhibit PhoR^S^ and PhoR^E^ autophosphorylation with more than 75% and more than 40% compared to the negative control, respectively (Fig. 3 and Table S3).

The F1 and F2 analogues inhibited WalK and PhoR^E^ autophosphorylation activity compared to the negative control from 11 to 62% and 17 to 80%, respectively (Fig. 2 and Table S4). Only F1.6 inhibited WalK autophosphorylation by more than 50% at a concentration of 2 mM meaning that the remaining 18 of the tested F1 and F2 analogues are weak (Ki ≫ 2 mM) WalK autophosporylation inhibitors. Inhibition of PhoR^E^ autophosphorylation was greater than 50% for F1.8, F2.1, F2.2, F2.8 and F2.9 meaning that the remaining 12 of the tested compounds are weak PhoR^E^ autophosphorylation inhibitors (Table S4).

The antibacterial effect evaluation of LBSS hits with two Gram-positive (*S. aureus* DSM20231 and *S. epidermidis* DSM 20044) and one Gram-negative (*E. coli* CFT 073) strains showed that S1.7 was bacteriostatic for *S. aureus* DSM 20231 with a MIC of 250 μg/ml (Table 2), S1.13 was bactericidal for *S. aureus* DSM 20231 and *S. epidermidis* DSM 20044 with MIC of 8 and 1 μg/ml, respectively, and MBCs of 33 and 8 μg/ml, respectively (Table 2). Additionally, S1.14 was bactericidal for *S. aureus* DSM 20231 and *S. epidermidis* DSM 20044 at a MIC and MBC of 500 μg/ml (Table 2). F1.6, F1.8, F2.3, F2.4 and F2.8 showed moderate antibacterial effects with MICs in the range of 31 to 250 μg/ml for *S. aureus* DSM 20231 and 31 to 500 μg/ml for *S. epidermidis* DSM 20044 (Table 2). Only F1.8 and F2.8 had antibacterial effect on the Gram negative *E. coli* CFT 073 with MICs < 250 μg/ml. With the exception of F2.2 and F2.9, those compounds showing HK autophosphorylation inhibitory activity also showed antibacterial activity (Tables 2, S3 and S4), suggesting the possibility that the antibacterial activity might be mediated through the inhibition of HK autophosphorylation.

### HK autophosphorylation is inhibited in a dose-dependent manner

The IC_50_ of the putative HK inhibitors (F2.2. and F2.9 were excluded since they did not show antibacterial effect, see below) were measured in a multiple concentrations at one time point (30 sec) kinase assays (Tables S3 and S4, Figs 2 and 3). F1.8 inhibited PhoR^E^ autophosphorylation with IC_50_ ≤ 1 mM (reduced solubility in kinase buffer at concentrations higher than 1 mM; Table S4, Fig. 2). F2.1 and F2.8 inhibited PhoR^E^ autophosphorylation with IC_50_ 0.24 and 0.72 mM, respectively, (Table S4, Fig. 2). F2.1 and F2.8 showed good solubility in kinase buffer in the presence of 10% DMSO. S1.7 inhibits PhoR^E^ autophosphorylation with IC_50_ in the lower micromolar range (≥100 μM) and PhoR^S^ in the higher micromolar/milimolar range (IC_50_ PhoR^S^ ≥ 1000 μM). The IC_50_ curves indicated that S1.7 is not soluble in concentrations higher than 1 mM in kinase buffer in the presence of 10% DMSO (Fig. 3, Table S3). S1.13 inhibits PhoR^E^ and PhoR^S^ with IC_50_ PhoR^E^ = 16 μM and IC_50_ PhoR^S^ = 212 μM and possess good solubility in kinase buffer in the presence of 10% DMSO (Fig. 3, Table S3). S1.14 IC_50_ against PhoR^E^ and PhoR^S^ is higher than 2000 μM and higher than 1000 μM, respectively (Fig. 3, Table S3).

### The putative HKAIs showed antibacterial effects against multi-drug resistant clinical isolates

Next, we studied the antibacterial effect of F1, F1.6, F1.8, F2.3, F2.4, F2.8, S1.7, S1.13 and S1.14 on a panel of clinical isolates and reference strains of pathogenic bacteria (Tables 2 and S5). The methicillin-resistant *S. aureus* (MRSA) strains are well characterized and are resistant to all β-lactam antibiotics and a range of non-β-lactam antibiotics^55^. *S. epidermidis* clinical isolates were obtained from wounds of patients admitted to Tawam Hospital (Al Ain, United Arab Emirates). The clinical isolates of the Gram-negative *Acinetobacter baumannii*^56^ and *Stenotrophomonas maltophilia*^57^ are well characterized and show multi-drug resistance. Additional reference strains included the Gram-positive *S. aureus* ATCC 25293, *S. epidermidis* RP62A and RP62A, *S. pneumoniae* 49619 and the emerging zoonotic pathogen *Streptococcus suis* 3881/S10, and the Gram-negative *E. coli* ATCC 25276, *Klebsiella pneumoniae* ATCC 700603 and *Pseudomonas aeruoginosa* ATCC 27853.

F1 and F1.6 (halogen-substituted phenyl-thiazoleamines) showed similar antibacterial activities for the panel of clinical isolates and reference strains. Both F1 and F1.6 showed antibacterial effect for the MRSA strains (with the exception of F1.6 for V4180 MRSA strain) and *S. aureus* 25293 with MICs in the range of 125–500 μg/ml (Table 2). V4180 MRSA strain is resistant to a wider range of antibiotics compared to the other MRSA strains tested (Table S5) including the small molecule antibiotics chloramphenicol and sulfamethaxazole. Given the broad range of antibiotic resistance of V4180 MRSA it is reasonable to propose that the presence of putative efflux pumps for small molecules could be responsible for the lack of susceptibility to F1.6. F1 and F1.6 showed antibacterial effect on one of the three tested *S. epidermidis* clinical isolates with MICs of 63 and 125 μg/ml, respectively. F1.6 MIC for *S. pneumoniae* 49619 was 256 μg/ml. F1 and F1.6 did not show antibacterial effect on *S. suis* 3881/S10 or on any of the Gram-negative strains tested.

F1.8 (bromophenyl-pyrimidinediamine) showed antibacterial effect on all the Gram-positive strains tested. F1.8 MICs for the *S. aureus* reference strains and the MRSA strains were in the range of 125 to 250 μg/ml. F1.8 MICs for the *S. epidermidis* strains were in the range of 31 to 500 μg/ml. F1.8 MICs for *S. suis* 3881/S10 and *S. pneumoniae* 49610 were 250 μg/ml and 128 μg/ml, respectively. F1.8 showed antibacterial effect for all the Gram-negative strains tested except for the *S. maltophilia* B32/1 strain. F1.8 MICs for the Gram-negative *A. baumannii* strains, *E. coli* ATCC 25276 and *K. pneumoniae* ATCC 700603 were in the range of 125–250 μg/ml and the MIC for *P. aeruoginosa* ATCC 27853 was 500 μg/ml. In a similar way to F1.6 with V4180 MRSA, the lack of susceptibility of *S. maltophilia* B32/1 to F1.8 could be explained by the presence of efflux pumps. *S. maltophilia* B32/1 is also resistant to the small-molecule β-lactam antibiotic, meropenem so that efflux pumps with broad substrate specificity may be involved^58^.

The MICs of F2.3, F2.4 and F2.8 (substituted carbazoles) for the MRSA strains and *S. aureus* 25293 were in the range of 125–500 μg/ml. F2.3 and F2.8 MICs for the *S. epidermidis* strains were in the range of 31–250 μg/ml. F2.3 and F2.4 MICs for *S. pneumoniae* 49610 were 4 μg/ml and 64 μg/ml, respectively. F2.4 and F2.8 MICs for *S. suis* 3881/S10 were 125 and 250 μg/ml, respectively. F2.3 and F2.8 did not show antibacterial effect for the *A. baumannii* and *S. maltophilia* strains. F2.4 MICs for three of the five *A. baumannii* strains and two of the three *S. maltophilia* strains tested were 500 μg/ml. F2.4 and F2.8 MICs for *E. coli* ATCC 25276 were 500 μg/ml. F2.8 MIC for *K. pneumoniae* ATCC 700603 was 250 μg/ml. None of F2.3, F2.4 and F2.8 showed antibacterial effect for *P. aeruginosa* ATCC 27853.

S1.7 (N-[3-(2-chlorophenyl)phenyl]acetamide) inhibited the growth of two of the six MRSA strains *in vitro* with MICs ≥250 μg/ml, which was comparable to the MICs for the *S. aureus* reference strains 25293 and DSM 202231. The other four MRSA strains were not susceptible to S1.7. As expected from the results of the antibacterial susceptibility testing with *S. epidermidis* DSM 20044, compound S1.7 had no antibacterial effect on the clinical isolates of *S. epidermidis*. S1.7 inhibits the growth of all tested *S. pneumoniae* strains with MIC of 128 μg/ml, which was similar to the MICs for the reference strains of *S. aureus*. S1.7 did not inhibit the growth of the *S. suis* 3881/ S10 strain in the tested concentration range (≤500 μg/ml). The strain specific antibacterial effects of S1.7 correlate well with the quite different IC_50_ observed for PhoR^E^ and PhoR^S^ HKs.

S1.13 (4-[(3S,4R)-4-(4-fluorophenyl)hexan-3-yl]phenol) inhibited the growth of all tested MRSA stains with MICs between 8 and 16 μg/ml (Table 2), which was comparable to the MICs for the reference strains of *S. aureus*. S1.13 also inhibited the growth of clinical isolates of *S. epidermidis* with MICs between 8 and 16 μg/ml, which is similar to the MICs for the biofilm forming *S. epidermidis* RP62A and non-biofilm forming *S. epidermidis* RP62A/1. Variation in MIC distributions for different strains of a species e.g. *S. aureus* is typical and reported^76^. Thus our finding that *S. epidermidis* 20044 has a MIC of 1 μg/ml and the rest of the tested *S. epidermidis* strains have MICs between 8 and 16 μg/ml is not unusual, but does indicate that some strains are more sensitive than others. When at least 100 strains are tested the MIC, which inhibits 50% of the isolates is typically considered the ‘intrinsic resistance’ level of wild type isolates. Further studies on the further development of these inhibitors will include MIC assays on hundreds of isolates to calculate MIC_50_ and MIC_90_ values. Like S1.7, S1.13 inhibited the growth of the *S. pneumoniae* strains with MIC of 16 μg/ml. S1.13 MICs against multi-drug resistant clinical isolates are comparable with the MICs against reference strains. This suggests that the putative mechanism of action of S1.13 differs from the known antibiotics and/or the mechanisms of resistance of the tested strains to known antibiotics are not functional against S1.13.

S1.14 (4-tert-butyl-2-[(phenylamino)methyl]phenol) inhibits growth of MRSA with MICs of 250 μg/ml, which was comparable to the MICs for the reference strains of *S. aureus*. S1.14 inhibited growth of all *S. epidermidis* strains except *S. epidermidis* strain RP62A, with MICs in the range of 250 to 500 μg/ml. S1.14 was not active against *S. pneumoniae* strains but inhibited growth of the *S. suis* S10 with MIC of 125 μg/ml. S1.14 inhibits also the growth of the Gram-negative *A. baumanii* and *S. maltohilia* strains with MICs from 250 μg/ml to 500 μg/ml. S1.7, S1.13 and S1.14 did not inhibit the growth of *K. pneumoniae* ATCC 700603 and *P. aeruginosa* ATCC 27853 at the highest tested concentration (MIC > 500 μg/ml).

### Increased antibacterial activity is not related to unspecific mechanism of action

**Figure 4:**
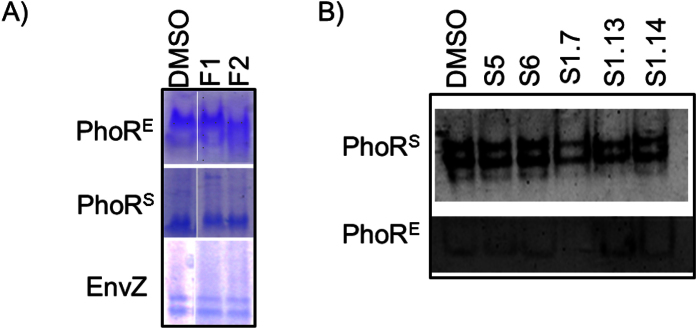
(**A**) F1 and F2 (2 mM) do not cause HK aggregation as demonstrated by native-PAGE with *E. coli* PhoR (PhoR^E^) and EnvZ, and *S. aureus* PhoR (PhoRS). (**B**) Compounds S5 and S6 (5 mM), and S1.7, S1.13 and S1.14 (2 mM) do not cause HK aggregation as demonstrated by native-PAGE with PhoR^S^ and PhoR^E^ HKs.

Protein aggregation and membrane damage have been described as mechanism of action of previously reported HKAIs^59^. To discard these mechanisms of action, protein aggregation activity of the hit compounds F1, F2, S5 and S6 was evaluated by native polyacrylamide gel electrophoresis using the catalytic portions (DHp and CA domains) of PhoR^E^ as a target. Neither inhibitor caused protein aggregation when the compounds were added in high (2 mM) concentration (Fig. 4). Furthermore, similar assays carried out with additional HKs (PhoR^S^ or EnvZ) or with the second generation compounds S1.7, S1.13 and S1.14, showed similar results (Fig. 4), suggesting that inhibitory activity of the compounds is unrelated with protein aggregation.

To check the potential of HK inhibitors to cause membrane damage^60^ hemolysis experiments with erythrocytes from a healthy donor were performed. Hemolysis was observed only with compound S1.13 at concentrations higher than the observed MICs (LC_50_ 277 μg/ml). The rest of the tested inhibitors (S1.7, S1.14, F1, F1.6, F1.8, F2.3, F2.4 or F2.8) did not cause erythrocyte hemolysis at 500 μg/ml (LC_50_ > 500 μg/ml) indicating that the inhibitors do not cause loss of integrity of the erythrocyte plasma membrane at their MICs.

### Inhibition of HK autophosphorylation is predicted to be mediated by interactions with key residues of the ATP-binding site of the HK CA domain

To get insights into the putative interaction mode of the reported HKAIs with the CA domains of HKs, molecular docking experiments were performed. The HKAIs (S5, S6, S1.7, S1.13, S1.14, F1, F1.6, F1.8, F2.3, F2.4, F2.8) and ADP as an internal control were docked to the CA domain of *T. maritima* HK853 (PDB: 3DGE) using the GOLD docking software^61^. The RMSD between the docked ADP and the cognate ADP structure in the HK853 was 1.0 Å. This corresponds to a successful re-docking calculation and, therefore, validates the used docking parameters. Due to low molecular weight of the HKAIs (Table S6), it is possible that the HKAIs possess more than one binding mode. Encouragingly, within the top 20 solutions for each HKAI only one or two binding modes were predicted. This together with the low RMSD for the docked ADP gave us confidence about the predicted binding modes of the reported putative HKAIs.

**Figure 5:**
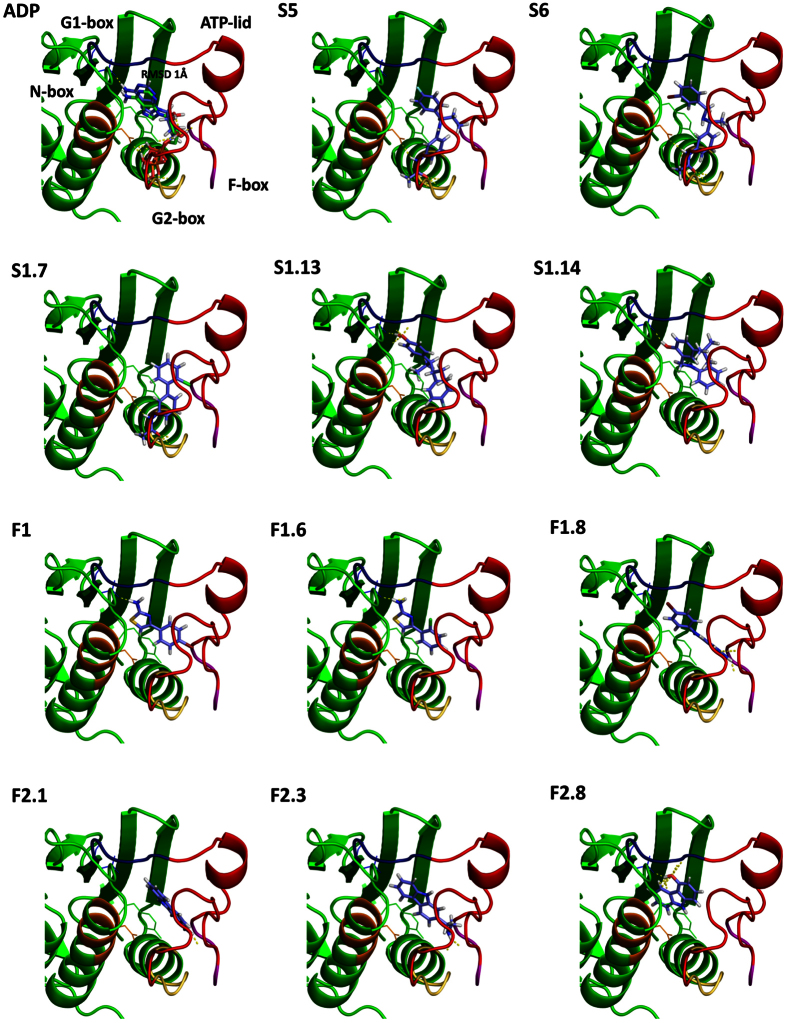
Predicted binding modes of selected HKAIs. All inhibitors (shown in blue as sticks) dock in the ATP-binding site of HK853 with a predicted binding mode resembling the experimental data (3DGE) from the natural product ADP (Top left). They interact with key elements involved in ATP-binding and autophosphorylation, i.e the N-, G1-, G2-boxes (shown in orange, blue and yellow, respectively) and the ATP-lid (shown in red).

A common feature for the predicted binding modes of all the compounds was the presence of an aromatic ring that accommodates into the hydrophobic cavity occupied by the pyrimidine ring of adenine (Fig. 5). The aromatic ring forms π–π stacking interactions with Y384 on one side and van der Waals contacts with I416 on the other side of the ring. Similar hydrophobic interactions have been observed for the adenine in the structures of *T. maritima* HK853 and other HKs in complex with nucleotides^62^,^32^. Located at the bottom of the ATP-binding hydrophobic pocket is the conserved Asp (D411) in the G1 box that gives specificity for recognition of the N6 amino group in the pyrimidine ring of adenine (Fig. 5). For S1.13, the most potent inhibitor, and S1.14, the hydroxyl group of the phenolic ring stacked in the adenine pocket is predicted to be hydrogen bonded to the conserved D411 residue. F1, F1.6, F1.8, F2.4, and F2.8 are also predicted to form polar contacts with D411 either by hydrogen bonds via classical hydrogen-donor groups (-OH, NH_2_) or by halogen atoms (e.g. Br, Fig. 5). Similar mode of interaction was observed for the binding of the Hsp90 inhibitor radicicol to the ATP-binding domain of the HK PhoQ^63^. The reported HKAIs are also predicted to interact with the ATP-lid, a variable loop involved in nucleotide binding and autophosphorylation^64^,^34^, and with other conserved elements in the ATP-binding site such as the N- and G2-boxes. S5, S6 and S1.7 are predicted to exploit their common amide moieties to mediate polar interaction on the part of the active site, which in HK structures with the native ligand is occupied by the nucleotide phosphates and the Mg^2+^ cation. In the predicted binding modes of S5, S6, and S1.7, the nitrogen of the amide moiety is predicted to be hydrogen bonded to the conserved N-box Mg^2+^ chelating residue N380 whereas the oxygen is predicted to bind to the main-chain of the G2-Box residues G443, L444 and G445, mimicking in this way the interactions of the ATP γ-phosphate^34^. In addition, the docking experiments also predict that F1, F1.8, F2.3, F2.4 form hydrogen bonds with the ATP-lid (Fig. 5).

Overall, the predicted binding modes of the selected HKAIs indicate that main HKAI-HK interactions are mediated by hydrophobic ring stacking that are mimicking the adenine of the cognate ligand. Additionally, stabilizing interactions are formed with binding site residues that are conserved and are crucial for nucleotide selection and autophosphorylation, including D411 in the G1-box and residues in the N and G2-boxes, and the ATP-lid. This supports the *in vitro* results and suggests that the HKAIs possess general HK autophosphorylation inhibitory activity and also could inhibit further HKs not tested in this study.

### S1.13 shows comparable *in vitro* activities to previously published HKAIs

**Figure 6:**
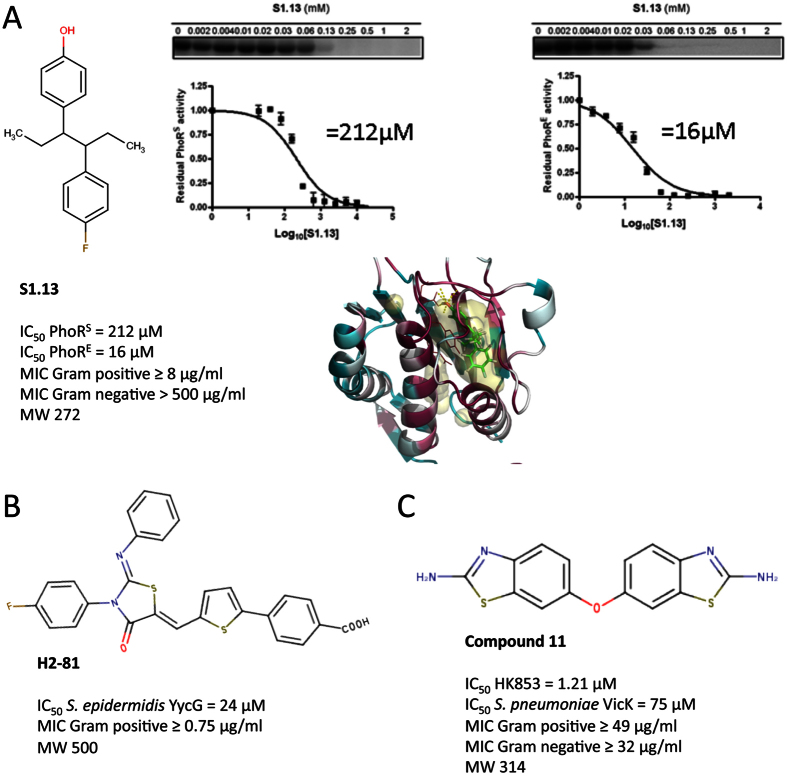
Comparison of S1.13 and previously reported HKAIs. (**A**) S1.13 inhibits the autophosphorylation of HK from a Gram positive (PhoR^S^) and a Gram negative (PhoR^E^) species with IC_50_ of 212 and 16 μM, respectively, shows antibacterial effect against Gram positive strains with MICs ≥ 8 μg/ml, and has a MW < 300, indicating a high potential for improvement. (**B**) H2-81 (MW 500) was derived from previously reported HKAI following rational design^65^, inhibits *S. epidermidis* YycG with IC_50_ = 24 μM, and shows antibacterial effect against Gram positive strains with MICs ≥ 0.75 μg/ml. (**C**) Compound 11 (MW 314) was identified in a high-throughput screening for HKAIs^9^, inhibits *T. maritima* HK853 and *S. pneumoniae* VicK autophosphorylation with IC_50_ of 1.21 μM and 75 μM, respectively, and shows antibacterial effect against both Gram positive and Gram negative bacteria with MICs ≥ 49 and MICs ≥ 32, respectively.

Structure-based virtual screenings for *S. epidermidis* WalK ligands yielded inhibitors, which were subsequently further optimized by rational design^65^. The last generation of *S. epidirimids* WalK inhibitors had MICs for *S. epidermidis* and *S. aureus* lower than 3.1 μM, corresponding to MIC lower than 1.66 μg/ml^65^. S1.13, the most potent HKAI reported here, possess MICs for *S. epidermidis* laboratory strains and clinical isolates in the range of 1 to 8 μg/ml. S1.13 MICs for reference strains of *S. aureus* and clinical isolates of MRSA are in the range from 8 to 16 μg/ml (Fig. 6, Table 2). Therefore, in terms of antibacterial activity S1.13 is comparable to the last generation of published *S. epidermidis* WalK inhibitors. The previous reported inhibitors showed IC_50_ values in the range of 24.2 to 71.2 μM for the inhibition *S. epidermidis* WalK autophosphorylation reaction^65^. S1.13 has not been tested for its autophosphorylation inhibitory capacity for any WalK, however, it inhibits PhoR^E^ and PhoR^S^ with IC_50_ of 16 and 212 μM, respectively (Fig. 6, Table 2). Considering the differences in the experiments used to determine the IC_50_s of the *S. epidermidis* WalK inhibitors and S1.13 and the particular catalytic constants in the autophosphorylation reaction for each HK (e.g. Km for ATP has been reported from 2 to 200 μM^33^), we can consider that all these inhibitors show a similar range (low micromolar) of autophosphorylation inhibitory activity. However, S1.13 (MW 272.4) is a relatively smaller compound compared to the last generation *S. epidermidis* WalK inhibitors (MW between 498.02 and 534.02) implying that it possesses higher ligand efficiency and more possibilities for further optimization (Fig. 6).

Recently, Wilke and collaborators^9^ have reported a HTS of 53000 compounds for HKAIs that yielded a number of putative HKAIs. The best hit in terms of *in vitro* activities, compound 11, inhibits HK853 and *S. pneumoniae* VicK autophosphorylation with IC_50_ of 1.21 and 75 μM, respectively (Fig. 6). The MICs for the Gram-positive *B. subtilis* and the Gram negative *E. coli* DC2 were in the range of 49–64 and 32–64 μg/ml respectively. S1.13 was not active against any Gram-negative strain tested (Table 2), however, the MICs against the Gram-positive strains tested were 2 to 8 times lower than the MIC of compound 11 for *B. subtilis*. The latter supports that structure-based and fragment-based approaches present an efficient way for generation of hits for putative HK autophosphorylation inhibitors that can be further used in antibacterial drug discovery following hit-to-lead optimization. Furthermore, S1.13 has been recently tested in the NCI/DTP One Dose/ 60 cell line screen (NCI-60)^52^. NCI-60 includes a collection of tumor cell lines derived from a variety of human adult cancer tissue types and is commonly used for genetic analysis and screening of potential chemotherapeutic agents. 2.72 μg/ml (10 μM) of S1.13 did not inhibit the growth of any of the cell lines with more than 19%, and most of the cell lines growth was not inhibited^66^. Finally, the analysis of inhibitors databases as well as the current bibliography indicated that the scaffold of S1.13, as well as the scaffolds of S1.7 and F1.8, had not been previously proposed as antibacterial agents, pointing to these compound as a promising starting point for the development of broad-spectrum antibacterials with polypharmacology effect.

### SBVS and *in vitro* screenings alone or in combination yield promising hits for antibacterial drug discovery

The growing problem of MDR has motivated efforts in antibacterial drug discovery in recent years and different essential and non-essential targets absent in mammals have been explored^67^. For example, the essential CDP-ME kinase (IspE) contributes to the non-mevalonate or deoxy-xylulose phosphate (DOXP) pathway for isoprenoid precursor biosynthesis found in many species of bacteria and apicomplexan parasites. Tidten-Luksch and collaborators employed a *in silico* and *in vitro* screenings against IspE to identify non-substrate like inhibitors^67^. The two strategies were complementary, delivering chemically distinct hits with *in vitro* biochemical activities in the high micromolar to low millimolar range for the *in silico* screening hits, and in the low micromolar range for the *in vitro* screening hits. The success rate (size of starting library/ number of hits for which IC_50_ could be measured) was < 0.01% for the *in silico* screening and 0.03% for the *in vitro* screening^67^. Similarly, the approach reported here to identify putative HKAIs comprising SBVS and FBS, followed by LBSS, yielded distinct scaffolds with IC_50_ in the micromolar to low milimolar range and antibacterial effect *in vitro*. The success rate for the SBVS was < 0.01%, and 0.2% for the FBS, confirming that different approaches result in comparable success rates in identifying hits for antibacterial drug discovery.

### Summary

Here we report the identification of putative bacterial HKAIs with broad-spectrum antibacterial effect against both Gram-positive and Gram-negative pathogens using a combination of *in silico* and *in vitro* screens. The most potent hit, S1.13, is bactericidal against Gram-positives, including multi-drug resistant MRSA, with MBCs ≤ 16 μg/ml. The MICs of S1.13 for Gram-positive bacteria are higher than the MICs of the recently published promising antimicrobial teixobactin (0, 06–4 μg/ml)^68^. Nevertheless, the much smaller molecular weight (S1.13 272.4 vs. teixobactin 1242.47), relatively simpler chemical structure, drug-like physicochemical properties, and the expected polypharmacology effect make S1.13 a very promising hit for development of novel antibacterials to treat Gram-positive infections. The antibacterial effects of the reported hits against *E. coli* (F1.8 ≥ 250 μg/ml and F2.8 ≥ 63 μg/ml) are negligible when compared to teixobactin (2–25 μg/ml). Both the reported HKAIs and teixobactin are not active against *P. aeruginosa* and *K. pneumoniae*. Nevertheless, HKAIs are targeted at intracellular targets, namely the CA domains of HKs, and were shown to inhibit the autophosphorylation of HKs from Gram-negative bacteria (i.e. PhoR^E^). This implies that their antibacterial activity against Gram-negatives could be improved by medicinal chemistry or delivery methods that facilitate passage of the inhibitor through the outer membrane. This would also make HKAIs a promising starting point for the development of antibacterials with polypharmacology against Gram-negatives. Teixobactin is proposed to interfere with one of the membrane-associated steps of peptidoglycan biosynthesis. Although resistance development to teixobactin has not been detected^68^, resistance to antimicrobials with similar mechanism of action (e.g. vancomycin) is well-known. In conclusion, the reported HKAIs show comparable *in vitro* activities to previously reported HKAIs and comparable future potential to recently discovered antimicrobials to be further developed as broad-spectrum antibacterials.

## Materials and Methods

### Reagents

A fragment library of 898 compounds (>95% purity) was purchased from Chem-X-Infinity (Romanville, France) and the individual compounds were stored at −80 °C at a concentration of 20 mM. For screening purposes, cocktails of 10 compounds at final concentration of 2 mM were prepared in a 96-well-plate. Re-supply of compounds F2 and F2.5 to F2.9 (Table S2) were purchased from Sigma-Aldrich (Spain). Re-supply of F1 was purchased from Apolo Scientific (United Kingdom). Compounds F1.1 to F1.10 and F2.1 to F2.3 (Table S2) were obtained from Developmental Therapeutics program of the National Cancer Institute and the National Institute of Health (DTP NCI/NIH)^52^. Compounds S1 to S10 from the initial SBVS screening were purchased from Ukrainian Organic Synthesis (Kiev, Ukraine). Compounds S1.1 to S1.25 from the ligand-based similarity searches (LBSS) were obtained from DTP. γ-32P-ATP was purchased from Perkin Elmer. Compounds were dissolved in 100% DMSO and stored at 4 °C protected from direct light. [γ-32] ATP was purchased from Perkin Elmer.

### Cloning, expression and purification

*S. pneumoniae walK* encoding the catalytic portion (DHp and CA domain) of WalK (amino acids from 208 to 449) was amplified by PCR from *S. pneumoniae* CDC3059-06 genomic DNA using the following primers: forward 5′-aagttctgtttcagggcccgatggagcaggagaaggaagaacgc-3′ and reverse 5′-atggtctagaaagctctagtcttctacttcatccac-3′. The PCR product was purified by PCR product purification kit (Macherey-Nagel) and cloned into a gel-purified pOpinF vector (kindly provided by Nick Berow, IRB, Spain) linearized with KpnI and HindIII (Fermentas). The insert was cloned into the pOpinF vector with InFusion HD cloning system (Clontech). Positive clones were confirmed by colony PCR and DNA sequencing.

*S. pneumoniae* WalK (WalK) was expressed in *E. coli* RIL. Luria Broth (LB) media supplemented with 100 μg/ml ampicillin and 33 μg/ml chloramphenicol was inoculated with an overnight pre-culture (1/50 of the culture volume). At exponential phase (OD600 0.2–0.4) protein expression was induced by addition of 1 mM IPTG for 3 to 5 h at 37 °C. The cells were harvested by centrifugation at 4000 g, 4 °C for 25 min and the pellets were stored at −80 °C until use. The cell pellets were resuspended in lysis buffer (100 mM Tris pH 8.0, 150 mM NaCl, 0.1 mM PMSF) and sonicated at 4 °C for 5 min at pulses of 15 sec every 1 minute The cell debris and the supernatant were separated by centrifugation at 11000 g, 4 °C for 60 min. The cell debris were resuspended in equilibration buffer (100 mM Tris pH 8.0, 150 mM NaCl) containing 2 M urea and incubated overnight at 4 °C with rotation. After centrifugation at 11 000 g, the supernatant was injected into a Ni-affinity chromatography column (GE Healthcare) equilibrated with equilibration buffer, washed with 5 volumes of equilibration buffer and eluted with equilibration buffer containing 0.5 M imidazole. WalK was concentrated with AmiconUltra (Millipore, USA) centrifugal filters, aliquoted and stored at −80 °C until use. The yield was ≤0.5 mg/L culture.

The catalytic portion (DHp and CA domain) of *E. coli* PhoR (PhoR^E^), *E. coli* EnvZ, and *S. aureus* PhoR (PhoR^S^), and the CA domain of *Synechococcus sp.* PCC 7942 NblS were expressed and purified as previously described^34^,^64^,^69^,^70^. Shortly, proteins were expressed in *E. coli* RIL and purified by Ni-affinity and size-exclusion chromatography. Purified proteins were stored in 20–50 μl aliquots at −80 °C.

### Structure-based virtual screening

#### Target preparation

The chosen molecular targets for molecular docking were the CA domains of *Thermotoga maritima* HK853 (PDB: 3DGE)^64^, *Geobacillus stearothermophillus* KinB (PDB: 3D36)^44^ and *T. maritima* CheA (PDB: I58B)^43^. Residues corresponding to the CA domain of each A chain (320–480 for 3DGE , 270–415 for 3D36 and 354–540 for I58B) were selected for each structure and additional atoms corresponding to water molecules, ions or ligands were removed. Hydrogen atoms were added in the absence of the cognate ligand using the GOLD program^61^.

#### Docking parameters

All docking calculations were performed with the GOLD docking software (version 5.2) using ChemPLP as a scoring function^49^. Binding sites were defined as being 10 Å around the geometric centre of the cognate ligand.

#### Library

For the initial screening, a diversity set (600 000) of the Scopius–CSpace database (over 6 million commercially available drug-like compounds)^71^ (http://inhibox.com), was docked into each of the three HK structures. The search efficiency parameter was set to 30%, 10 solutions were generated for each compound of which only the highest-scoring poses were saved.

#### Post-processing of docking results

Compounds with unwanted functional groups (in-house rules used by InhibOx) were removed and the resulting set of compounds was ranked in two lists: i) by the ChemPLP GOLD docking score (ChemPLP) and ii) by a ligand efficiency score (l.e.) which is ChemPLP divided by the number of non-hydrogen atoms in the compound^50^. The top 3500 compounds in each list were used to extract the top 100 compounds docking to all three HKs CA domain structures. This resulted in two final lists of compounds: one with respect to ChemPLP and one with respect to ligand efficiency. The top 100 compounds of each list were finally visually inspected and ten compounds were purchased for experimental testing.

### Ligand-based similarity searches

The database from the Developmental Therapeutics program of the National Cancer Institute and the National Institute of Health (DTP)^52^ was searched for analogue structures of the initial hits F1, F2, S5 and S6. The similarity searches with F1, F2, S5 and S6 as query molecules were performed using the Morgan fingerprint as implemented in RDKit^54^, which is a variation of the “extended connectivity fingerprints” (ECFP)^53^. The top 100 hits of each similarity search were visually inspected of which in total 25 compounds were ordered and experimentally tested.

### Binding mode prediction

Docking calculations to predict the binding modes of the reported fragments were performed using the ATP-binding domain of *T. maritima* HK853 (PDB: 3DGE, chain A, residues from 270 to 415) and the GOLD docking software^61^. For each ligand 100 solutions were generated of which the top 20 were visually inspected. In Fig. 5 the dominant binding mode within the top 20 solutions is shown for the respective ligand.

### Differential scanning fluorimetry

To monitor protein unfolding, the fluorescent dye Sypro orange was used^26^. Differential scanning fluorimetry (DSF) experiments were conducted in the iCycleriQ Real Time Detection System (Bio-Rad, Hercules, CA). Solutions of 20 μl of 0.1 mg/ml protein (final concentration), 200 μM fragment cocktails or individual fragments (final concentration), 10X sypro orange (final concentration) and buffer (100 mM TrisCl pH 8, 150 mM NaCl) were added to the wells of the 96-well iCycler iQ PCR plate. The plate was heated from 20 to 85 or 99 °C at a heating rate of 1 °C/min. The fluorescent intensity was measured with Ex/Em: 490/530 nm. Prism GraphPad v.5 was used for curve fitting and statistical analysis^72^.

### Kinase assay

To evaluate the inhibitory capacity of selected hits from DSF, SBVS and LBSS *in vitro* autophosphorylation kinase assays with γ-^32^P-ATP were performed as previously described^62^. Ligands were dissolved in 100% DMSO. When comparing the inhibitory capacity of ligands in one concentration-one time point experiments or when measuring IC_50_ (the concentration at which 50% residual HK autophosphorylation activity is observed), the final DMSO concentration in the assays was 10% (v/v). Controls lacking ligands contained an equal concentration of DMSO. Inhibition of autophosphorylation was determined by incubating 0.12 mg/ml (≈4 μM) HK and up to 20 mM fragment in kinase buffer (50 mM Tris HCl, pH 8.5, 50 mM KCl, 5 mM MgCl2, 0.5 mM EDTA and 0.1 mM DTT). Autophosporylation reactions were initiated by addition of 0.1 μCi/μl γ-^32^P-ATP containing from 0.03 to 0.06 μM ATP (final concentrations). Autophosphorylation was quenched with 2xSDS-PAGE sample buffer supplemented with 50 mM EDTA. Samples were applied without heating to 15% (w/v) Tris-glycine SDS-polyacrylamide gels. After electrophoresis, the bottoms of the gels were removed to lower the background from the unincorporated radiolabeled ATP. Gels were dried without staining on a Bio-Rad Gel Air drying system and the phosphorylated protein was quantified by phosphor-imaging. Prism GraphPad was used for curve fitting and statistical analysis^72^.

### Aggregation analysis by native polyacrylamide gel electrophoresis

*E. coli* PhoR and EnvZ, and *S. aureus* PhoR (0.12 μg/ml, final concentration) were prepared in kinase buffer. Compounds were added to a final concentration of 5 mM (S5 and S6) or 2 mM (F1 and F2, S1.7, S1.13, S1.14). DMSO in the assays was maintained to a final concentration 10% (v/v). After 30 min of incubation at room temperature Native polyacrylamide gel electrophoresis (Native-PAGE) loading buffer was added and samples loaded. Coomassie blue staining was used for protein visualization.

### Antibacterial susceptibility testing

Bacterial strains used in this study for antibacterial susceptibility testing (Table S5) were propagated using standard microbiological procedures. Minimal inhibitory concentrations (MICs) were determined following a standard double-dilution method^73^. MICs were recorded as the lowest concentration of the compound where no visible growth was observed. After plating the dilutions around the MIC or growing them in fresh MH media, minimal bactericidal concentration (MBC) was recorded as the lowest concentration of the compound at which no colonies were formed or no growth was observed, respectively. For *S. pneumoniae* MICs were determined by adapting the standard double-dilution method to anaerobic conditions and of this microorganism (use of Todd Hewitt Yeast extract with 200 U/mL of catalase and continuous monitoring of growth). MBCs for *S. pneumoniae* were determined by inoculation of 10 μl from each well that did not shown visible bacterial growth on THY 0,5% 3% blood agar plates. After 24 h of incubation at 37 °C 5% CO_2_, the first dilution yielding three colonies or fewer was scored as the MBC, as described by the CLSI for starting inoculate of 1 × 10^5^ CFU/ml^74^.

### Hemolysis assay

Hemolytic activity against human erythrocytes taken from a healthy donor was measured as previously described^73^. The hemolysis experiments were carried out in accordance with the guidelines of the United Arab Emirates University (UAEU) Research Ethics Review Board. All experimental protocols were approved by the Research Ethics Review Board of UAEU and informed consent was obtained from all donors.

Erythrocytes were incubated with (up to) 500 μg/ml compounds and the LC_50_ value was recorded as the mean concentration of compound producing 50% hemolysis in three independent incubations.

## Additional Information

**How to cite this article**: Velikova, N. *et al*. Putative histidine kinase inhibitors with antibacterial effect against multi-drug resistant clinical isolates identified by *in vitro* and *in silico* screens. *Sci. Rep.* **6**, 26085; doi: 10.1038/srep26085 (2016).

## Supplementary Material

Supplementary Information

## Figures and Tables

**Table 1:** Selected compounds from the FBSS and SBVS and their corresponding IC_50_ and MICs.

**Initial hits**					**MIC μg/ml**		
	**IC**_**50**_ **[mM]**				***S. aureus***	***S. epidermidis***	***E. coli***
**Name**	**PhoR**^**S**^	**PhoR**^**E**^	**HK853**	**WalK**	**DSM 20231**	**DSM 20044**	**CFT 073**
F1	n.d.	2	n.d.	>2	25	4	>500
F2	n.d.	0.3	n.d.	>2	31	>500	>500
S1	>5	>5	>5	>5	>500	>500	>500
S2	>5	>5	>5	>5	>500	>500	>500
S3	>5	>5	>5	>5	>500	>500	>500
S4	>5	>5	>5	>5	>500	>500	>500
S5	≈1	≈1	>5	>1	>500	500^#^	>500
S6	1.14	0.37	>5	>1	>500	500^#^	500^#^
S7	<5*	>5	>5	>5	>500	>500	>500
S8	<5*	>5	>5	>5	>500	>500	>500
S9	>5	>5	>5	>5	>500	>500	>500
S10	>5	>5	>5	>5	>500	>500	>500

^*^% Inhibition at 5 mM ≥ 70%, i.e. IC50 < 5 mM.

^#^MBC > 500 μg/ml.

**Table 2:** Antibacterial activities of selected HKAIs for a panel of clinical isolates and reference strains.

**Strain**	**MIC (μg/ml)**								
	**F1**	**F1.6**	**F1.8**	**F2.3**	**F2.4**	**F2.8**	**S1.7**	**S1.13**	**S1.14**
*Staphylococcus aureus*									
20231	25	125	125	125	250	31	250	8	500
25293	125	250	250	125	125	250	125	8	125
274/08	125	500	250	>500	500	250	250	16	250
V4180	125	>500	250	250	500	250	>500	8	250
T4/6	125	250	250	250	>500	250	250	8	250
145/08	125	250	250	250	250	250	>500	8	250
127/08	n.d	250	250	250	500	250	>500	16	250
S908	n.d	500	250	250	500	n.d.	>500	16	250
*Staphylococcus epidermidis*									
20044	4	500	63	31	500	31	>500	1	500
RP62A	>500	>500	500	125	> 500	250	>500	8	>500
RP62A/1	250	>500	250	125	n. d.	250	>500	8	500
T7/3	63	125	31	31	n. d.	63	>500	8	250
T37/8	>500	>500	500	125	n. d.	250	>500	16	500
T6119	>500	>500	250	125	n. d.	250	>500	8	500
*Streptococcus suis*									
3881/S10	>500	>500	250	>500	250	125	>500	8	125
*Streptococcus pneumoniae*									
49619	n. d.	256	128	4	64	n. d.	128	16	>500
*Acinetobacter baumannii*									
NM109	n. d.	n. d.	125	n. d.	500	n. d.	>500	>500	250
NM124	n. d.	n. d.	125	n. d.	500	n. d.	>500	>500	250
NM8	>500	>500	125	>500	500	>500	>500	>500	500
NM35	n. d.	n. d.	125	n. d.	>500	n. d.	>500	>500	500
NM75	n. d.	n. d.	250	n. d.	>500	n. d.	>500	>500	500
*Stenotrophomonas maltophilia*									
B5/5	n. d.	n. d.	n. d.	n. d.	>500	n. d.	>500	>500	500
B6/2	n. d.	n. d.	n. d.	n. d.	500	n. d.	>500	>500	500
B32/1	>500	>500	>500	>500	500	>500	>500	>500	500
*Escherichia coli*									
CFT 073	>500	>500	250	>500	500	63	>500	>500	>500
ATCC 25276	>500	>500	250	>500	500	500	>500	>500	>500
*Klebsiella pneumoniae*									
ATCC700603	>500	>500	250	>500	>500	250	>500	>500	>500
*Pseudomonas aeruginosa*									
ATCC 27853	>500	>500	500	>500	>500	>500	>500	>500	>500

n.d.–not tested.
