# Supplementary material for: Putative histidine kinase inhibitors with antibacterial effect against multi-drug resistant clinical isolates identified by in vitro and in silico screens
Source: Sci Rep. 2016 May 13;6:26085. doi: 10.1038/srep26085 (PMC4865847; doi:10.1038/srep26085)
Supplement: Supplementary Information [file srep26085-s1.doc]

Putative histidine kinase inhibitors with antibacterial effect against multi-drug resistant clinical isolates identified by *in vitro* and *in silico* screens

Nadya Velikova, Simone Fulle, Ana Sousa Manso, Milena Mechkarska, Paul Finn, J. Michael Conlon, Marco Rinaldo Oggioni, Jerry M. Wells, Alberto Marina

Table of Contents:

Supplementary Figures 2-6

Supplementary Tables 7-13

References 13


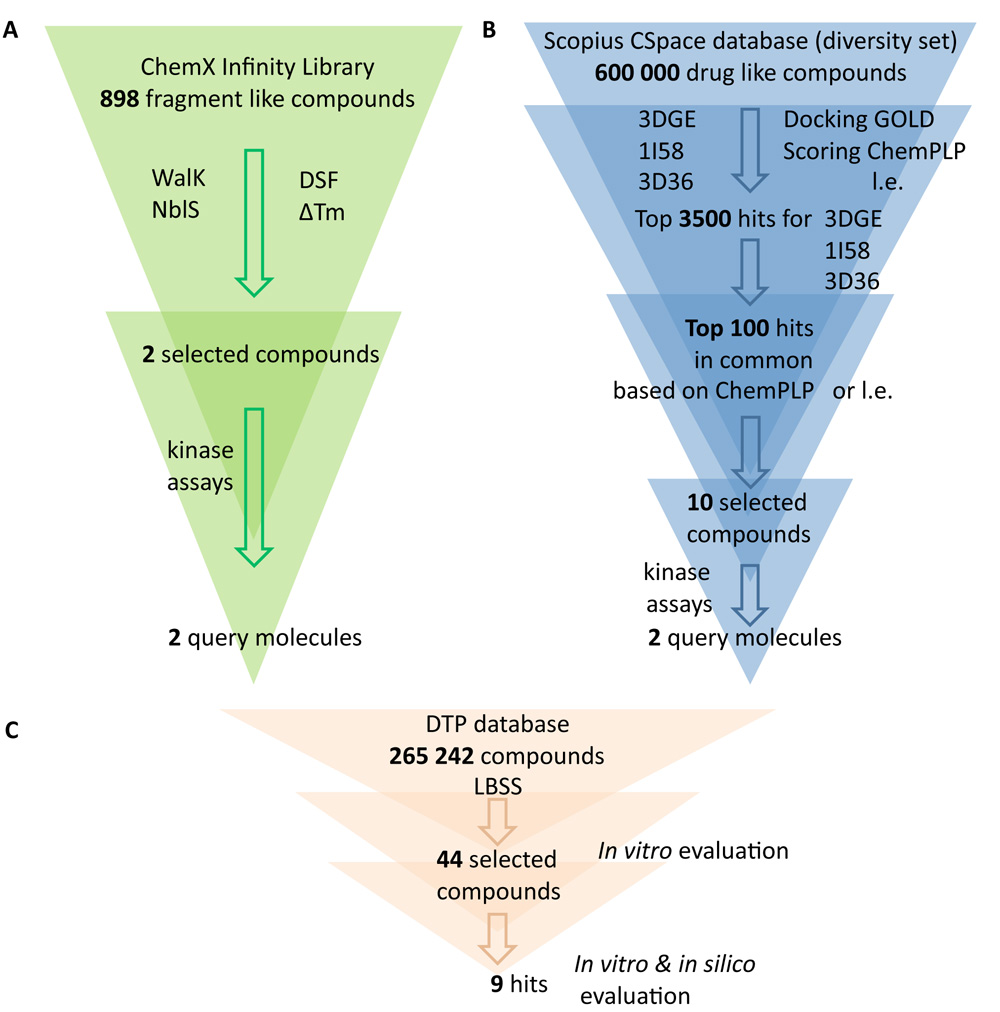


**Figure S1**. Identification of putative histidine kinase autophosphorylation inhibitors (HKAIs) with antibacterial effect by *in silico* and *in vitro* screens. A) Fragment-based screening (FBS). In the *in vitro* FBS a fragment library was screened for ligands of both the CA domain of *Synechococcus sp*. PCC 7942 NblS and CA and DHp domains of *Streptococcus pneumoniae* WalK. The two hits in common were used as query molecules in LBSS. B) Structure-based virtual screening (SBVS). In the SBVS a diversity set of the Scopius CSpace database was screened for putative ligands of the ATP-binding site of the CA domain of three HK structures using GOLD. Out of the top 3500 hits for each HK, the top 100 in common for the three HKs based on ChemPLP or ligand efficiency (l.e.) were visually inspected and 10 compounds were selected for experimental testing. Based on the results of the *in vitro* evaluation by kinase assay and antibacterial susceptibility 2 hits were used as query molecules in ligand-based similarity searches (LBSS). C) LBSS of the National Cancer Institute Developmental Theraupeutics Programme Database (DTP) with the hits identified by SBVS and FBS as query molecules resulted in 44 compounds selected for experimental testing of which 9 further evaluated *in vitro* and *in silico*.

**Figure S2**. Identification of F1 (D4.1) and F2 (G5.9) as putative CA ligands by DSF. The fragment-library was divided in cocktails of ten fragments, each at a final concentration of 2 mM in 96-well plates. Each plate included a negative control (DMSO) and positive-controls (ADP, ATP, AMP-PNP). A) The individual compounds from the cocktails which increased the Tm of NblS (A) and the Tm of WalK (D) were tested. F1 (D4.1) and F2 (G5.9) were identified as the compounds present in the D4 and G5 cocktails that most likely caused the observed increase of NblS and WalK Tm (B, C, E, F).


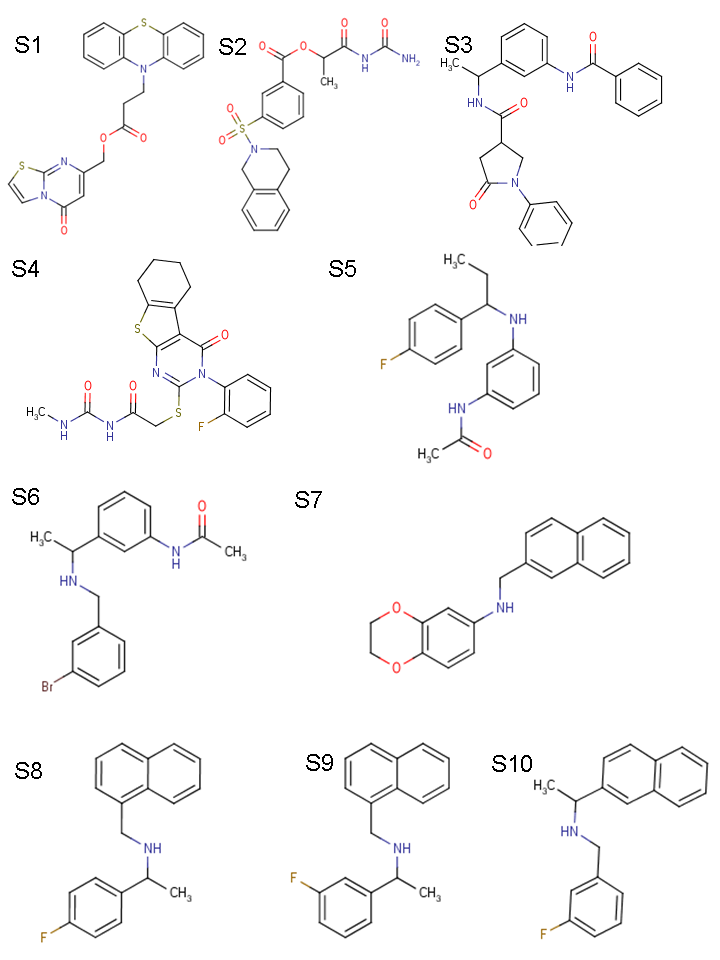


**Figure S3** Chemical structures of the hits from the structure-based virtual screening (S1-10) selected for experimental testing.


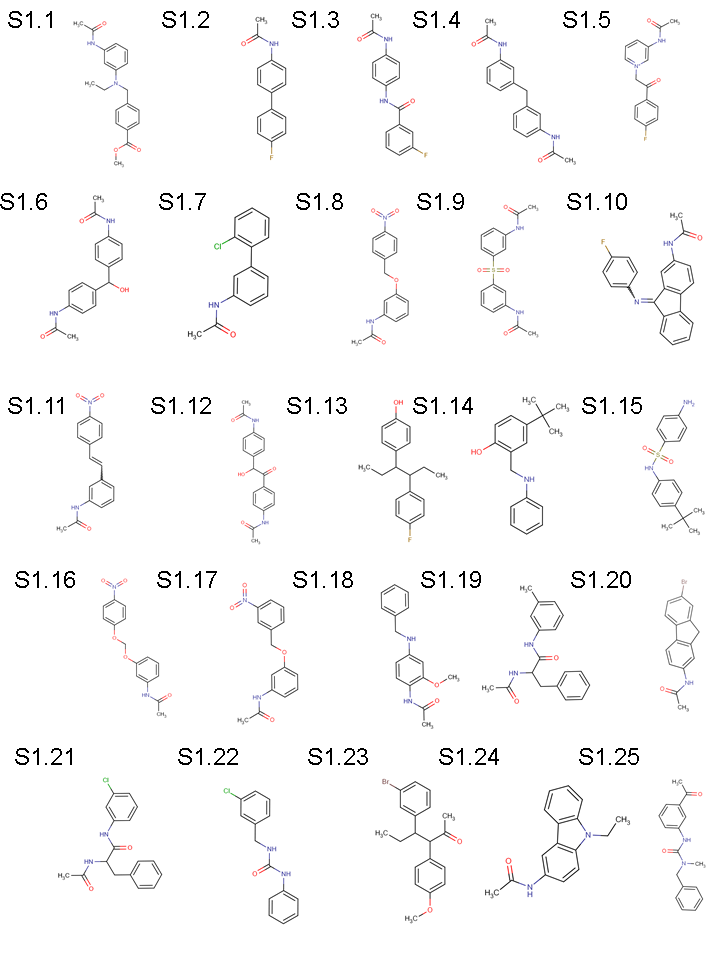


**Figure S4.** Chemical structures of the analogues of the SBVS hits, S5 and S6, selected for experimental testing following ligand-based similarity searches (LBSS). The results of the *in vitro* evaluation by kinase assay and antimicrobial susceptibility testing are presented in Table S4.

**Figure S5**. Chemical structures of the analogues of FBS hits, F1 and F2, selected for experimental testing following LBSS. The results of the *in vitro* evaluation by kinase assay and antimicrobial susceptibility testing are presented in Table S5.

Table S1. Compounds selected from SBVS and their corresponding ChemPLP and ligand efficiency.

| Initial hits | Docking score^¥^ | |  |
| --- | --- | --- | --- |
| Name | ChemPLP | Ligand efficiency |  |
|  |  |  |  |
| ADP | 85.4 | 3.16 |  |
| S1 | 95.12 | 3.171 |  |
| S2 | 96.14 | 3.214 |  |
| S3 | 93.48 | 2.918 |  |
| S4 | 97.21 | 3.24 |  |
| S5 | 82 | 3.905 |  |
| S6 | 80.38 | 3.828 |  |
| S7 | 84.21 | 3.828 |  |
| S8 | 80.11 | 3.815 |  |
| S9 | 81.97 | 3.903 |  |
| S10 | 82.15 | 3.912 |  |
| ¥ | The values for HK853 (PDB: 3DGE, chain A) are presented | |  |

Tabe S2. Selected compounds for experimental testing from SBVS (S1 to S10), DSF (F1 and F2) and LBSS (S1.1 to S1.25, F1.1 to F1.10 and F2.1 to F2.9)

| Name | Provider´s Name | Smile |
| --- | --- | --- |
| S1 | PB-02322356 | O=C(CCN1C2=C(SC3=C1C=CC=C3)C=CC=C2)OCC1=CC(=O)N2C=CSC2=N1 |
| S2 | PB-04723907 | CC(OC(=O)C1=CC(=CC=C1)S(=O)(=O)N1CCC2=C(C1)C=CC=C2)C(=O)NC(N)=O |
| S3 | PB404910184 | CC(NC(=O)C1CN(C(=O)C1)C1=CC=CC=C1)C1=CC(NC(=O)C2=CC=CC=C2)=CC=C1 |
| S4 | PB-06200251 | CNC(=O)NC(=O)CSC1=NC2=C(C3=C(CCCC3)S2)C(=O)N1C1=C(F)C=CC=C1 |
| S5 | BBV-129624 | CCC(NC1=CC(NC(C)=O)=CC=C1)C1=CC=C(F)C=C1 |
| S6 | BBV-174972 | CC(NCC1=CC(Br)=CC=C1)C1=CC(NC(C)=O)=CC=C1 |
| S7 | BBV-129155 | C(NC1=CC2=C(OCCO2)C=C1)C1=CC2=C(C=CC=C2)C=C1 |
| S8 | BBV-34226801 | CC(NCC1=C2C=CC=CC2=CC=C1)C1=CC=C(F)C=C1 |
| S9 | BBV-34226813 | CC(NCC1=C2C=CC=CC2=CC=C1)C1=CC(F)=CC=C1 |
| S10 | BBV-203039 | CC(NCC1=CC(F)=CC=C1)C1=CC2=C(C=CC=C2)C=C1 |
| S1.1 | 310269 | CCN(CC1=CC=C(C=C1)C(=O)OC)C2=CC(=CC=C2)NC(C)=O |
| S1.2 | 73090 | CC(=O)NC1=CC=C(C=C1)C2=CC=C(F)C=C2 |
| S1.3 | 214040 | CC(=O)NC1=CC=C(NC(=O)C2=CC(=CC=C2)F)C=C1 |
| S1.4 | 86683 | CC(=O)NC1=CC(=CC=C1)CC2=CC(=CC=C2)NC(C)=O |
| S1.5 | 400630 | CC(=O)NC1=C[N+](=CC=C1)CC(=O)C2=CC=C(F)C=C2 |
| S1.6 | 4288 | CC(=O)NC1=CC=C(C=C1)C(O)C2=CC=C(NC(C)=O)C=C2 |
| S1.7 | 109741 | CC(=O)NC1=CC(=CC=C1)C2=C(Cl)C=CC=C2 |
| S1.8 | 211557 | CC(=O)NC1=CC(=CC=C1)OCC2=CC=C(C=C2)[N](=O)=O |
| S1.9 | 85756 | CC(=O)NC1=CC(=CC=C1)[S](=O)(=O)C2=CC(=CC=C2)NC(C)=O |
| S1.10 | 12323 | CC(=O)NC1=CC2=C(C=C1)C3=CC=CC=C3C2=NC4=CC=C(F)C=C4 |
| S1.11 | 211552 | CC(=O)NC1=CC(=CC=C1)C=CC2=CC=C(C=C2)[N](=O)=O |
| S1.12 | 106213 | CC(=O)NC1=CC=C(C=C1)C(O)C(=O)C2=CC=C(NC(C)=O)C=C2 |
| S1.13 | 32652 | CCC(C(CC)C1=CC=C(F)C=C1)C2=CC=C(O)C=C2 |
| S1.14 | 48154 | CC(C)(C)C1=CC(=C(O)C=C1)CNC2=CC=CC=C2 |
| S1.15 | 7436 | CC(C)(C)C1=CC=C(N[S](=O)(=O)C2=CC=C(N)C=C2)C=C1 |
| S1.16 | 211556 | CC(=O)NC1=CC(=CC=C1)OCOC2=CC=C(C=C2)[N](=O)=O |
| S1.17 | 107560 | CC(=O)NC1=CC(=CC=C1)OCC2=CC(=CC=C2)[N](=O)=O |
| S1.18 | 118968 | COC1=C(NC(C)=O)C=CC(=C1)NCC2=CC=CC=C2 |
| S1.19 | 408367 | CC(=O)NC(CC1=CC=CC=C1)C(=O)NC2=CC(=CC=C2)C |
| S1.20 | 68235 | CC(=O)NC1=CC2=C(C=C1)C3=CC=C(Br)C=C3C2 |
| S1.21 | 408365 | CC(=O)NC(CC1=CC=CC=C1)C(=O)NC2=CC(=CC=C2)Cl |
| S1.22 | 205456 | ClC1=CC=CC(=C1)CNC(=O)NC2=CC=CC=C2 |
| S1.23 | 130858 | CCC(C(C(C)=O)C1=CC=C(OC)C=C1)C2=CC(=CC=C2)Br |
| S1.24 | 67710 | CC[N]1C2=C(C=CC=C2)C3=C1C=CC(=C3)NC(C)=O |
| S1.25 | 205718 | CN(CC1=CC=CC=C1)C(=O)NC2=CC=CC(=C2)C(C)=O |

Table S2. (continued) Selected compounds for experimental testing from SBVS (S1 to S10), DSF (F1 and F2) and LBSS (S1.1 to S1.25, F1.1 to F1.10 and F2.1 to F2.9)

| **Name** | **Provider´s name** | **Smile** |
| --- | --- | --- |
| **F1 (D4.1)** | **OR5604** | **NC1=NC(=CS1)C2=CC=C(Br)C=C2** |
| **F1.1** | **NSC 405294** | **NC1=NC(=CS1)C2=CC=C(O)C=C2** |
| **F1.2** | **NSC 372682** | **NC1=NC(=CS1)C2=CC=C(Cl)C=C2** |
| **F1.3** | **NSC 54436** | **CC1=CC=C(C=C1)C2=CSC(=N2)N** |
| **F1.4** | **NSC 176404** | **NC1=NC=C(S1)C2=CC=C(Br)C=C2** |
| **F1.5** | **NSC 614448** | **NC1=NN=C(O1)C2=CC=C(Br)C=C2** |
| **F1.6** | **NSC 13534** | **NC1=NC(=CS1)C2=C(Cl)C=CC=C2** |
| **F1.7** | **NSC 223276** | **BrC1=CC=C(C=C1)C2=CSC3=NCCN23** |
| **F1.8** | **NSC 80819** | **NC1=NC(=CC=N1)NC2=CC=C(Br)C=C2** |
| **F1.9** | **NSC 329206** | **COC1=C(OC)C=C(C=C1)C2=CSC(=N2)N** |
| **F1.10** | **NSC 206952** | **CC(C)(C)C1=CC=C(OCC2=C[NH]C=N2)C=C1** |
| **F2 (G5.9)** | **213497 ALDRICH** | **OC1=CC2=C(C=C1)C3=CC=CC=C3[NH]2** |
| **F2.4** | **NSC 305336** | **CC(C)NCC(O)COC1=C2C(=CC=C1)[NH]C3=CC=CC=C23** |
| **F2.1** | **NSC 171107** | **NC1=CC2=C(C=C1)C3=CC=CC=C3[NH]2** |
| **F2.2** | **NSC 402750** | **OC1=CC2=C([NH]C3=C(C=CC=C3)[C]2=O)C=C1** |
| **F2.3** | **NSC 106510** | **NCCC1=CC2=C(C=C1)C3=CC=CC=C3[NH]2** |
| **F2.5** | **C5132 SIGMA** | **[NH]1C2=CC=CC=C2C3=CC=CC=C13** |
| **F2.6** | **325325 ALDRICH** | **C[N]1C2=CC=CC=C2C3=CC=CC=C13** |
| **F2.7** | **I3408 ALDRICH** | **[NH]1C=CC2=CC=CC=C12** |
| **F2.8** | **543896 ALDRICH** | **OC1=CC=CC2=C1C3=CC=CC=C3[NH]2** |
| **F2.9** | **754781 ALDRICH** | **CCC1=CC2=C([NH]C3=CC=CC=C23)C=C1** |

Table S3 Autophosphorylation inhibitory activities and antibacterial activities of compounds S1.1-S1.25

|  | IC50 [mM] | | MIC [µg/ml] | | |
| --- | --- | --- | --- | --- | --- |
| Name | PhoR^S^ | PhoR^E^ | *S. aureus* | *S. epidermidis* | *E. coli* |
|  |  |  | DSM 20231 | DSM 20044 | CFT 073 |
| S1.1 | > 2 | > 2 | >500 | >500 | >500 |
| S1.2 | <2 | <2 | >500 | >500 | >500 |
| S1.3 | > 2 | > 2 | >500 | >500 | >500 |
| S1.4 | > 2 | > 2 | >500 | >500 | >500 |
| S1.5 | > 2 | > 2 | >500 | >500 | >500 |
| S1.6 | > 2 | > 2 | >500 | >500 | >500 |
| S1.7 | ≈ 1 | ≈ 0.1 | 250^#^ | >500 | >500 |
| S1.8 | > 2 | > 2 | >500 | >500 | >500 |
| S1.9 | > 2 | > 2 | >500 | >500 | >500 |
| S1.10 | > 2 | > 2 | >500 | >500 | >500 |
| S1.11 | <2 | <2 | >500 | >500 | >500 |
| S1.12 | > 2 | > 2 | >500 | >500 | >500 |
| S1.13 | 0.212 | 0.016 | 8^¥^ | 1^¥^ | >500 |
| S1.14 | 1.48 | > 2 | 500^¶^ | 500^¶^ | >500 |
| S1.15 | <2 | > 2 | >500 | >500 | >500 |
| S1.16 | > 2 | > 2 | >500 | >500 | >500 |
| S1.17 | > 2 | > 2 | >500 | >500 | >500 |
| S1.18 | > 2 | > 2 | >500 | >500 | >500 |
| S1.19 | > 2 | > 2 | >500 | >500 | >500 |
| S1.20 | > 2 | > 2 | >500 | >500 | >500 |
| S1.21 | > 2 | > 2 | >500 | >500 | >500 |
| S1.22 | > 2 | > 2 | >500 | >500 | >500 |
| S1.23 | > 2 | > 2 | >500 | >500 | >500 |
| S1.24 | > 2 | > 2 | >500 | >500 | >500 |
| S1.25 | > 2 | > 2 | >500 | >500 | >500 |

^#^ S7 *S. aureus* MBC > 500 µg/ml

^¥^ S1.13 MBC *S. aureus* DSM20231 31 µg/ml; S1.13 MBC *S. epidermidis* DSM20044 8 µg/ml

^¶^ S1.14 MBC *S. aureus* DSM20231 and S1.14 MBC *S. epidermidis* DSM20044 500 µg/ml

Table S4. Autophosphorylation inhibitory activities and antibacterial activities of F1, F1.1 – F1.10, F2, and F2.1 to F2.9

|  | IC50 [mM] | | MIC [µg/ml] | | | MBC [µg/ml] | | |
| --- | --- | --- | --- | --- | --- | --- | --- | --- |
| Name | PhoR^E^ | WalK | *S. aureus* | *S. epidermidis* | *E. coli* | *S. aureus* | *S. epidermidis* | *E. coli* |
|  |  |  | DSM 20231 | DSM 20044 | CFT 073 | DSM 20231 | DSM 20044 | CFT 073 |
| F1 (D4.1) | ≈ 2 | > 2 | 25 | 4 | >500 | >500 | 250 | n.d. |
| F1.1 | > 2 | > 2 | 500 | >500 | 500 | >500 | n.d | >500 |
| F1.2 | > 2 | > 2 | >500 | >500 | >500 | n.d. | n.d | n.d. |
| F1.3 | > 2 | > 2 | >500 | 500 | >500 | n.d. | 500 | n.d. |
| F1.4 | > 2 | > 2 | >500 | >500 | >500 | n.d. | n.d | n.d. |
| F1.5 | > 2 | > 2 | >500 | >500 | >500 | n.d. | n.d | n.d. |
| F1.6 | > 2 | < 2 | 125 | 500 | >500 | 250 | 500 | n.d. |
| F1.7 | > 2 | > 2 | >500 | >500 | >500 | n.d. | n.d | n.d. |
| F1.8 | ≤ 1 | > 2 | 125 | 63 | 250 | 250 | 125 | 500 |
| F1.9 | > 2 | > 2 | >500 | >500 | >500 | n.d. | n.d | n.d. |
| F1.10 | > 2 | > 2 | >500 | >500 | >500 | n.d. | n.d | n.d. |
| F2 (G5.9) | 0.3 | > 2 | 31 | >500 | >500 | >500 | n.d. | n.d. |
| F2.4 | > 2 | > 2 | 250 | 500 | 500 | 250 | 500 | 500 |
| F2.1 | 0.24 | > 2 | 8 | >500 | >500 | >500 | n.d. | n.d. |
| F2.2 | < 2 | > 2 | >500 | >500 | >500 | n.d. | n.d. | n.d. |
| F2.3 | > 2 | > 2 | 125 | 31 | >500 | >500 | 63 | n.d. |
| F2.5 | > 2 | > 2 | >500 | >500 | >500 | n.d. | n.d. | n.d. |
| F2.6 | > 2 | > 2 | >500 | >500 | >500 | n.d. | n.d. | 500 |
| F2.7 | > 2 | > 2 | 500 | >500 | 500 | >500 | n.d. | n.d. |
| F2.8 | 0.72 | > 2 | 31 | 31 | 63 | 63 | 250 | 500 |
| F2.9 | < 2 | > 2 | >500 | >500 | >500 | >500 | n.d. | n.d. |

n.d. – not tested

Table S5 Bacterial strains.

| Strain | Source | Resistance |
| --- | --- | --- |
| *Staphylococcus aureus* DSM 20231 | DSMZ – German Collection of Microorganisms and Cell culture | Control strain |
| *Staphylococcus aureus* 25293 | ATCC | Control strain |
| *Staphylococcus aureus* 274/08 ^1^ | Tawam Hospital, Al Ain, UAE | β-lactams, Ka, Ne, C, E* |
| *Staphylococcus aureus* V4180 ^1^ | Tawam Hospital, Al Ain, UAE | β-lactams, A, G, Ka, Ne, S, Sxt, Tet, C, Rif, E, Cl* |
| *Staphylococcus aureus* T4/6 ^1^ | Tawam Hospital, Al Ain, UAE | β-lactams, Ka, Ne, C, E, Cl* |
| *Staphylococcus aureus* 145/08 ^1^ | Tawam Hospital, Al Ain, UAE | β-lactams, Ka, Ne, S, Tet, Fu* |
| *Staphylococcus aureus* 127/08 ^1^ | Tawam Hospital, Al Ain, UAE | β-lactams |
| *Staphylococcus aureus* S908 ^1^ | Tawam Hospital, Al Ain, UAE | β-lactams, C, E, Cl* |
| *Staphylococcus epidermidis* DSM 20044 | DSMZ – German Collection of Microorganisms and Cell culture | Control strain |
| *Staphylococcus epidermidis* RP62A (biofilm producer) | 2 | Control strain |
| *Staphylococcus epidermidis* RP62A/1(non-biofilm producer) ^2^ | 2 | Control strain |
| *Staphylococcus epidermidis* T7/3 | Tawam Hospital, Al Ain, UAE |  |
| *Staphylococcus epidermidis* T37/8 | Tawam Hospital, Al Ain, UAE |  |
| *Staphylococcus epidermidis* T6119 | Tawam Hospital, Al Ain, UAE |  |
| *Streptococcus* suis 3881/ S10 ^3^ | CVI, Lelystad |  |
| *Streptococcus pneumoniae* 49619 | ATCC |  |
| *Acinetobacter baumannii* NM109 ^4^ | Hospitals in Abu Dhabi Emirate, UAE | All antibiotics commonly used to treat *Acinetobacter* infections except colistin |
| *Acinetobacter baumannii* NM124 ^4^ | Hospitals in Abu Dhabi Emirate, UAE | All antibiotics commonly used to treat *Acinetobacter* infections except colistin |
| *Acinetobacter baumannii* NM8 ^4^ | Hospitals in Abu Dhabi Emirate, UAE | All antibiotics commonly used to treat *Acinetobacter* infections except colistin |
| *Acinetobacter baumannii* NM35 ^4^ | Hospitals in Abu Dhabi Emirate, UAE | All antibiotics commonly used to treat *Acinetobacter* infections except colistin |
| *Acinetobacter baumannii* NM75 ^4^ | Hospitals in Abu Dhabi Emirate, UAE | All antibiotics commonly used to treat *Acinetobacter* infections except colistin |
| *Stenotrophomonas maltophilia* B5/5 ^5^ | Tawam Hospital, Al Ain, UAE | meropenem |
| *Stenotrophomonas maltophilia* B6/2 ^5^ | Tawam Hospital, Al Ain, UAE | meropenem |
| *Stenotrophomonas maltophilia* B32/1 ^5^ | Tawam Hospital, Al Ain, UAE | meropenem |
| *Escherichia coli* CFT 073 | ATCC | Control strain |
| *Escherichia coli* 25276 | ATCC | Control strain |
| *Klebsiealla pneumoniae* 700603 | ATCC | Control strain |
| *Pseudomonas aeroginosa* 27853 | ATCC | Control strain |

*A, [amikacin](javascript:void(0);); C, [ciprofloxacin](javascript:void(0);); Cl, [clindamycin](javascript:void(0);); E, [erythromycin](javascript:void(0);); Fu, [fusidic acid](javascript:void(0);); G, gentamycin; Ka, [kanamycin](javascript:void(0);); Ne, [neomycin](javascript:void(0);); Rif, rifampicin; S, [streptomycin](javascript:void(0);); Sxt, [sulfamethoxazole](javascript:void(0);)+[trimethoprim](javascript:void(0);); Tet, tetracyline.

Table S6. Physicochemical properties of selected HKAIs

| HKAI | logS | logP | logD | MW | HBD | HBA | TPSA | Flexibility | Rotatable Bonds |
| --- | --- | --- | --- | --- | --- | --- | --- | --- | --- |
| S1.7 | 2.34 | 3.567 | 3.567 | 245.7 | 1 | 2 | 29.1 | 0.1667 | 3 |
| S1.13 | 0.9023 | 5.315 | 5.315 | 272.4 | 1 | 1 | 20.23 | 0.2381 | 5 |
| S1.14 | 1.664 | 4.263 | 2.333 | 255.4 | 2 | 2 | 32.26 | 0.2 | 4 |
| F1 | 2.85 | 2.61 | 1.775 | 255.1 | 1 | 2 | 38.91 | 0.07143 | 1 |
| F1.6 | 3.033 | 2.45 | 2.047 | 210.7 | 1 | 2 | 38.91 | 0.07143 | 1 |
| F1.8 | 2.447 | 2.154 | 1.258 | 265.1 | 2 | 4 | 63.83 | 0.125 | 2 |
| F2.3 | 3.077 | 2.459 | 1.054 | 210.3 | 2 | 2 | 41.81 | 0.1111 | 2 |
| F2.4 | 2.511 | 3.59 | 1.479 | 298.4 | 3 | 4 | 57.28 | 0.25 | 6 |
| F2.8 | 2.565 | 2.675 | 2.675 | 183.2 | 2 | 2 | 36.02 | 0 | 0 |

**References**

1 Sonnevend, A. *et al.* Change in meticillin-resistant Staphylococcus aureus clones at a tertiary care hospital in the United Arab Emirates over a 5-year period. *J Clin Pathol* **65**, 178-182, doi:10.1136/jclinpath-2011-200436 (2012).

2 Ziebuhr, W. *et al.* A novel mechanism of phase variation of virulence in Staphylococcus epidermidis: evidence for control of the polysaccharide intercellular adhesin synthesis by alternating insertion and excision of the insertion sequence element IS256. *Mol Microbiol* **32**, 345-356 (1999).

3 Wang, Y. *et al.* First report of the multiresistance gene cfr in Streptococcus suis. *Antimicrob Agents Chemother* **57**, 4061-4063, doi:10.1128/AAC.00713-13 (2013).

4 Sonnevend, A. *et al.* Characteristics of epidemic and sporadic strains of Acinetobacter baumannii isolated in Abu Dhabi hospitals. *J Med Microbiol* **62**, 582-590, doi:10.1099/jmm.0.055681-0 (2013).

5 Jumaa, P. A. *et al.* The molecular epidemiology of Stenotrophomonas maltophilia bacteraemia in a tertiary referral hospital in the United Arab Emirates 2000-2004. *Ann Clin Microbiol Antimicrob* **5**, 32, doi:10.1186/1476-0711-5-32 (2006).
